# Intracorporeal versus Extracorporeal Anastomosis for Laparoscopic Right Hemicolectomy: Short-Term Outcomes

**DOI:** 10.3390/jcm10245967

**Published:** 2021-12-19

**Authors:** Antonio Biondi, Gianluca Di Mauro, Riccardo Morici, Giuseppe Sangiorgio, Marco Vacante, Francesco Basile

**Affiliations:** 1 Department of General Surgery and Medical-Surgical Specialties, University of Catania, Via S. Sofia 78, 95123 Catania, Italy; abiondi@unict.it (A.B.); rimorici75@gmail.com (R.M.); pep.sang7@gmail.com (G.S.); fbasile@unict.it (F.B.); 2 Unit of General Surgery, University Hospital Policlinico-San Marco, 95123 Catania, Italy; gianluca.dimauro@asp.rg.it

**Keywords:** colorectal surgery, colorectal cancer, anastomosis, right hemicolectomy, intracorporeal anastomosis

## Abstract

Laparoscopic right hemicolectomy represents an effective therapeutic approach for right colon cancer (RCC). The primary aim of this study was to evaluate bowel function recovery, length of hospital stay, operative time, and the number of general and anastomosis-related postoperative complications from intracorporeal anastomosis (ICA) vs. extracorporeal anastomosis (ECA); the secondary outcome was the number of lymph nodes retrieved. This observational study was conducted on 108 patients who underwent right hemicolectomy for RCC; after surgical resection, 64 patients underwent ICA and 44 underwent ECA. The operative time was slightly longer in the ICA group than in the ECA group, even though the difference was not significant (199.31 ± 48.90 min vs. 183.64 ± 35.80 min; *p* = 0.109). The length of hospital stay (7.53 ± 1.91 days vs. 8.77 ± 3.66 days; *p* = 0.036) and bowel function recovery (2.21 ± 1.01 days vs. 3.45 ± 1.82 days; *p* < 0.0001) were significantly lower in the ICA group. There were no significant differences in postoperative complications (12% in ICA group vs. 9% in ECA group), wound infection (6% in ICA group vs. 7% in ECA group), or anastomotic leakage (6% in ICA group vs. 9% in ECA group). We did not observe a significant difference between the two groups in the number of lymph nodes collected (19.46 ± 7.06 in ICA group vs. 22.68 ± 8.79 in ECA group; *p* = 0.086). ICA following laparoscopic right hemicolectomy, compared to ECA, could lead to a significant improvement in bowel function recovery and a reduction in the length of hospital stay in RCC patients.

## 1. Introduction

Colon cancer is the third most frequently diagnosed cancer in both sexes, and right colon cancers (RCCs) are often detected at an advanced stage [1]. Laparoscopic right hemicolectomy represents an effective therapeutic approach for RCC. However, the technique for laparoscopic right hemicolectomy has not been definitively standardized due to concerns regarding the creation of an anastomosis [2]. Recent studies have found some advantages of intracorporeal anastomosis (ICA), compared to extracorporeal anastomosis (ECA), that include fewer postoperative complications, fewer conversions to open surgery, and a shorter length of hospitalization [3,4,5].

The most frequent postoperative complications following laparoscopy for RCC include anastomotic leakage (AL), postoperative bleeding, and wound infection [6]. AL, which is defined as a defect of the intestinal wall at the anastomotic site, is characterized by communication between the intra- and extraluminal compartments [7]. The rates of AL may vary from 30% to less than 3% depending on the type, technique, and site of surgery [8]. AL represents a major cause of postoperative mortality and morbidity, and the occurrence of AL may increase the risk of local recurrence and the need for reintervention, thus having a greater impact on the quality of life [9].

A prospective, multicentric international study conducted on 2515 patients who underwent elective or emergency right hemicolectomy or ileocecal resection showed that the overall AL rate was 7.4% (180/2444), the 30-day morbidity rate was 38.0% (*n* = 956), and the mortality rate was 2.6% (*n* = 66). Patients with AL showed a significantly increased mortality rate (10.6% vs. 1.6% no-leak patients; *p* > 0.001). AL was associated with a longer duration of surgery (OR = 1.007 per min; *p* = 0.0037), an open approach (OR = 1.9; *p* = 0.0037), and a stapled anastomosis (OR = 1.5; *p* = 0.041) [10]. Another prospective, multicentric study of 1102 patients (Anastomotic Leak After Colon Resection for Cancer—ANACO study) showed that preoperative nutritional status and the stapled anastomotic procedure represented the only independent risk factors of AL following surgery for RCC. Furthermore, the mortality risk was increased in older patients with low preoperative nutritional statuses, whereas laparoscopic procedures decreased postoperative morbidity [11,12].

Wound infection, which is the most common nosocomial infection in surgical patients, showed an incidence rate of 5–30%, and was associated with prolonged postoperative hospitalization and an increase in morbidity and costs [13]. Risk factors for wound infection include male sex, advanced age, previous chemotherapy, conversion from laparoscopic to open technique, reintervention within the first 30 postoperative days, and AL [14]. Other postoperative complications may include ileus, wound dehiscence, incisional hernia, impaired renal function, respiratory complications, and urinary retention. The main goal of pre- and postoperative care, if properly conducted, is to avoid the onset of such complications [15,16,17].

The primary aim of this study was to evaluate bowel function recovery, length of hospital stay, operative time, and the number of general and anastomosis-related postoperative complications from ICA vs. ECA; the secondary outcome was the number of lymph nodes retrieved.

## 2. Materials and Methods

We analyzed data from a total of 108 consecutive patients (48 men and 60 women), who were enrolled in the study from January 2014 to May 2020; of these, 64 underwent ICA and 44 underwent ECA. The study was conducted at two surgical centers in Sicily (Italy): the General Surgery Unit of the “Vittorio Emanuele” Hospital in Catania, and the Unit of General Surgery of the “Ospedale Civile” Hospital in Ragusa. Approval from the ethical committee was obtained. This study was performed according to the Strengthening the Reporting of Observational Studies in Epidemiology (STROBE) Statement [18].

**Figure 1:**
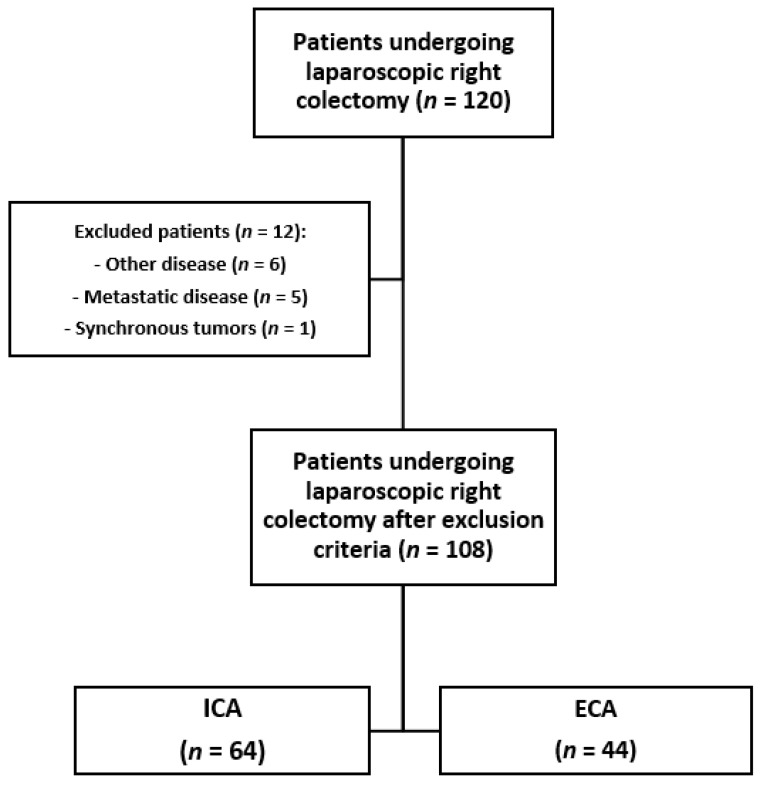
Study flowchart. ICA: Intracorporeal anastomosis; ECA: extracorporeal anastomosis.

We assessed patients with RCC, from stage I to stage III, according to the American Joint Committee on Cancer/Union for International Cancer Control (AJCC/UICC-TNM) classification [19], staged by colonoscopy with biopsy and total body computed tomography (CT) scan. Patients undergoing minimally invasive colectomy for RCC were included in the study. Exclusion criteria included open surgery or conversion to open surgery. We also excluded patients with unresectable metastases and/or tumors infiltrating nearby organs, candidates for palliative resections, and patients with synchronous tumors. All procedures were performed by 4 senior surgeons at our institution; all surgeons received their training at the University of Catania (Italy) and their skills were comparable. ECA or ICA was performed according to the clinical advice of each surgeon (Figure 1).

In the ECA group, after establishing a pneumoperitoneum, the intestine was exteriorized through an incision of about 6–10 cm in the right subcostal or median periumbilical region. A protection device was applied to protect the skin, muscles, and aponeurotic structures. Resection of the right colon was then performed, and the specimen was removed. Isoperistaltic side-to-side anastomosis was performed using a 60 mm linear stapler. The staple line was inspected for bleeding and small bleeding points were sutured with 000 silk figure-of-eight sutures. The anastomotic line was reinforced with a continuous absorbable monofilament (3-0 Vicryl), and the bowel was returned to the abdominal cavity. The incision was closed with interrupted or running sutures. Port-sites greater than 5 mm were closed with sutures. In the ICA group, the resection of transverse colon and terminal ileum were performed intracorporeally. Ileocolic side-to-side isoperistaltic anastomosis was performed using a 60 mm linear stapler, and then reinforced with continuous double-layer suture using 3-0 Vicryl. Bowel preparation was not used, and antibiotic prophylaxis was discontinued within the first 24 h. Drains were not routinely used, and the nasogastric tube was removed with extubation [20,21,22].

The following surgical and outcome parameters were assessed: operative time, length of hospital stay, bowel function recovery, complications related to anastomosis, wound infection, and number of lymph nodes collected. We collected data on demographics and cancer-related variables, including age, sex, ASA class, previous abdominal surgeries, and TNM staging. Follow up was 30 days. The study was conducted in accordance with the 1975 Declaration of Helsinki [23]. Informed consent was obtained from all participants included in the study.

### Statistical Analysis

Continuous variables were compared using Student’s *t*-test for independent samples. Chi-square test (or Fisher’s test when appropriate) was used for categorical variables. Statistical significance was assumed for *p* < 0.05. Statistical analysis was performed using SPSS 15.0 for Windows (IBM, Chicago, IL, USA).

## 3. Results

The two groups were demographically comparable; there were no significant differences in age, body mass index (BMI), American Society of Anesthesiologists (ASA) class, previous abdominal surgery, tumor localization, and stage of the disease (according to the AJCC/UICC TNM) (Table 1).

The operative time (199.31 ± 48.90 min vs. 183.64 ± 35.80 min; *p* = 0.109) was slightly higher in the ICA group than in the ECA group; however, this difference was statistically not significant. The length of hospital stay (7.53 ± 1.91 vs. 8.77 ± 3.66 days; *p* = 0.036) and bowel function recovery (2.21 ± 1.01 days vs. 3.45 ± 1.82 days; *p* < 0.0001) were significantly lower in the ICA group. We observed a similar number of lymph nodes collected in the two groups (19.46 ± 7.06 in ICA group vs. 22.68 ± 8.79 in ECA group; *p* = 0.086) (Table 2). Finally, we compared the differences in postoperative complications (12% of patients in ICA group vs. 9% of patients in ECA group), wound infection (6% of patients in ICA group vs. 7% of patients in ECA group), and AL (6% of patients in ICA group vs. 7% of patients in ECA group) (*p* = 0.856) (Table 3).

## 4. Discussion

Many studies have been conducted on ICA and ECA techniques for right hemicolectomy, analyzing short-term and long-term outcomes. In general, no significant differences between ICA and ECA were observed in mortality, and ICA proved to be a safe and reliable technique [24]. A recent observational study by Vallribera et al. reported higher overall morbidity in patients with ECA as compared to ICA (23.5% vs. 40.2%, *p* = 0.014; 5.9% vs. 14.9%, *p* = 0.039, respectively). Furthermore, no significant differences were observed in AL (9.8% vs. 10.3%, *p* = 0.55). These results suggested that ICA could reduce the risk for complications including AL, with only a minimal risk for wound complications [25]. The study by Vallribera et al. did not investigate the differences in the number of lymph nodes collected between the two anastomotic techniques, as we did. Nonetheless, the results of our study confirmed that the difference in AL between ICA and ECA was not significant; on the contrary, we did not observe significant differences in postoperative complications, such as abscesses, bleeding, and postoperative ileus (*p* = 0.366) (Table 3).

A recent multicentric randomized clinical trial (The IVEA-study) conducted on 168 patients who underwent laparoscopic right hemicolectomy for RCC showed that ICA reduced postoperative pain (*p* = 0.000), incision size (*p* = 0.000), and surgical site infection (3.65% vs. 16.67%, *p* = 0.008) compared to ECA [26]. Allaix et al. observed an earlier recovery of postoperative bowel function following ICA compared to ECA (gas: 2 (interquartile range—IQR 2–3) vs. 3 (IQR 2–3) days, *p* = 0.003; stool: 4 (IQR 3–5) vs. 4.5 (IQR 3–5) days, *p* = 0.032); however, no significant differences were reported in the median length of hospital stay (6 (IQR 5–7) vs. 6 (IQR 5–8) days; *p* = 0.839), number of lymph nodes collected, 30-day morbidity (17.1% vs. 15.7%, *p* = 0.823), length of skin incision, reoperation rate, or readmission rate [27].

Previous studies found that the number of lymph nodes collected could represent a prognostic factor after surgery for CRC [28,29,30]. A recent study showed a significant association between lymph node yields and survival outcomes in stage I and II CRC patients. A lymph node harvest of 20 or more was associated with better survival outcomes, whereas a lymph node harvest of less than 12 did not show inferior survival outcomes compared to those between 12 and 19 [31]. We did not observe significant differences between the two techniques in the number of lymph nodes collected (19.46 ± 7.06 in ICA group vs. 22.68 ± 8.79 in ECA group; *p* = 0.086).

Our results also showed a significant difference between ICA and ECA in terms of days needed for the return of bowel function (2.21 ± 1.01 in ICA group vs. 3.45 ± 1.82 in ECA group; *p* = 0.0001) and the length of hospital stay (7.53 ± 1.91 in ICA group vs. 8.77 ± 3.66 in ECA group; *p* = 0.036). These findings could be explained by the minor mobilization of the mesentery and digestive tract, in turn leading to an earlier recovery of intestinal functions. Furthermore, a smaller incision could decrease the incidence of postoperative pain, respiratory complications, and length of hospitalization [32,33]. According to numerous studies, the operative time for ICA is longer than for ECA, due to greater technical difficulties [34,35,36]; however, our study did not show a significant difference (199 ± 48.90 min in ICA group vs. 183.64 ± 35.80 min in ECA group; *p* = 0.109).

Finally, surgical complications associated with both major abdominal surgery and anastomosis technique were assessed. Our data did not show significant differences between the two techniques. AL was observed in 4/64 patients (6%) of the ICA group compared to 3/44 patients (7%) of the ECA group. These results are in line with the conclusions of several previous studies and confirmed the non-inferiority of ICA. A recent meta-analysis of 24 studies showed a significant reduction in ICA compared to ECA following laparoscopic right colectomy for both benign and malignant diseases, in parietal abscesses (OR 0.526, CI: 0.333–0.832, *p* = 0.006), time to first gas and stools, surgical repair, and length of hospitalization, with comparable general complications [37].

In our study, all anastomoses were performed using a linear stapler. A recent observational, retrospective study by Espin et al. conducted on 961 patients who underwent elective surgery for RCC demonstrated that the clinical impact of AL in patients with handsewn anastomosis (HA) was significantly lower than in patients with stapled anastomosis (SA) (*p* = 0.007). Indeed, patients with SA showed more severe complications and needed more re-laparotomies compared to patients with HA (*p* = 0.004). The authors did not find significant differences in the length of hospital stay of the patients with AL depending on the type of anastomosis (*p* = 0.275), and mortality due to AL was comparable between both groups [38]. Another prospective, observational, international, multicentric study investigated the relationship between the stapling technique (cutting vs. non-cutting stapler) and anastomotic failure following right hemicolectomy and ileocecal resection on 1347 patients. The study did not detect any difference in AL rates regarding the type of stapling device used to close the apical aspect. Furthermore, oversewing of the anastomotic staple lines did not show advantages in terms of reducing leak rates. A higher leak rate following surgery performed by general surgeons compared to by colorectal surgeons was observed [39]. A study conducted on 3208 patients who underwent right-sided colonic resection for malignancy or Crohn’s disease showed that patients undergoing HA were more likely to be emergency admissions (20.5% handsewn vs. 12.9% stapled) and to undergo open surgery (54.7% vs. 36.6%); SA was also associated with an increased AL rate [40]. These findings highlight the importance of developing a standardized type of anastomosis in order to improve therapeutic outcomes in patients undergoing right hemicolectomy.

Our study has some limitations: firstly, we evaluated only short-term outcomes in a small number of patients; secondly, ECA or ICA was performed according to the clinical advice of each surgeon. However, all operations were conducted by a team of surgeons using standardized operative techniques in comparable groups of CRC patients, which led to the achievement of consistent results.

## 5. Conclusions

Based on the results of our study, laparoscopic right hemicolectomy with ICA represents a feasible and safe technique. Compared to ECA, ICA displayed non-inferiority in terms of the operative time, incidence of complications, and number of lymph nodes removed, and superiority in terms of early bowel recovery function and reduction in the length of hospital stay. Further, well-designed clinical trials are needed to investigate possible applications of ICA in order to improve postoperative outcomes in RCC patients.

## Figures and Tables

**Table 1:** Demographic and clinical characteristics of the study population.

Variable	ECA (*n* = 44)	ICA (*n* = 64)	*p*-Value
Age (mean ± SD)	66.2 ± 12.6	68.0 ± 8.0	0.803
Male/Female (n)	26/18	37/27	0.894
BMI (Kg/m^2^) (mean ± SD)	25.0 ± 2.9	25.3 ± 3.0	0.602
ASA class			
I, *n* (%)	10 (23)	19 (30)	0.540
II, *n* (%)	26 (59)	32 (50)	0.611
III, *n* (%)	8 (18)	13 (20)	0.821
Previous surgery, *n* (%)	2 (4)	8 (12)	0.198
Stage (T)			
T1, *n* (%)	11 (25)	14 (22)	0.151
T2, *n* (%)	6 (14)	11 (17)	0.660
T3, *n* (%)	26 (59)	35 (55)	0.100
T4, *n* (%)	1 (2)	4 (6)	0.379
Stage (*N*)			
N0, *n* (%)	29 (66)	48 (75)	0.339
N1, *n* (%)	12 (27)	14 (22)	0.093
N2, *n* (%)	3 (7)	2 (3)	0.074

SD: standard deviation; *n*: number; BMI: body mass index; ASA: American Society of Anesthesiologists.

**Table 2:** Surgical outcomes.

	ECA (*n* = 44)	ICA (*n* = 64)	*p*-Value
Operative time (minutes)	183.64 ± 35.80	199.31 ± 48.90	0.109
Bowel function recovery (days)	3.45 ± 1.82	2.21 ± 1.01	<0.0001
Length of hospital stay (days)	8.77 ± 3.66	7.35 ± 1.91	0.036
Number of lymph nodes collected	22.68 ± 8.79	19.46 ± 7.06	0.086

**Table 3:** Complications after surgical treatment.

	*n*/ECA	% ECA	*n*/ICA	% ICA	*p*-Value
Postoperative complications	4/44	9%	8/64	12%	0.856
Abscess	0	0%	2	3%	
Bleeding	1	2%	0	0%	
Postoperative ileus	3	7%	6	9%	
Wound infection	3/44	7%	4/64	6%	0.366
Anastomotic leakage	3/44	7%	4/64	6%	0.366

*n*: number.

## Data Availability

The data presented in this study are available on request from the corresponding author.
